# Ligaplants: Uprising Regimen in the Glebe of Implant Dentistry

**DOI:** 10.7759/cureus.45968

**Published:** 2023-09-25

**Authors:** Pavan Bajaj, Unnati Shirbhate, Sneha Dare

**Affiliations:** 1 Department of Periodontics, Sharad Pawar Dental College, Datta Meghe Institute of Higher Education and Research, Wardha, IND

**Keywords:** biomaterial, tissue engineering, implants, tissue-engineered ligament, osseointegration, pdl, ligaplants

## Abstract

A dental implant is an alloplastic framework inserted into the bone, either straight through the alveolar bone or beneath the mucosa or periosteum, to support and hold a permanent or removable dental prosthesis. Osseointegration is a striking phenomenon in which bone directly opposes the implant surface without any interposing collagen or fibroblastic matrix. Although titanium metallic implants were the subject of "osseointegration" at first, it is now used to refer to any biomaterial that can osseointegrate. The science of tissue engineering allows for regenerating complete biological components outside the body for possible replacement treatment or therapy. It uses cells, organic or synthetic scaffold materials, and bioactive molecules. The combination of periodontal ligament (PDL) cells with implant biomaterial is known as Ligaplants. When placed in regions with significant periodontal bone defects, ligaplants can promote the development of new bone. PDL implants, inserted into the missing teeth extraction socket, facilitate surgery. To protect the PDL cell cushion, ligaplants are fitted initially loosely. However, they firmly integrate without interlocking or making direct contact with the bones. Osseointegrated implants affixed directly to the alveolar bone encircling them cannot serve the same purpose as healthy teeth because natural periodontal tissue deteriorates over time. To create a biological connection capable of performing specific physiological tasks, a tissue-engineered PDL must be constructed in conjunction with a dental implant that is well thought out.

## Introduction and background

Dental implants are artificial tooth roots. Implants can support permanent (fixed) or removable replacement teeth that are made to match your natural teeth precisely. One method of replacing lost teeth is with a dental implant. They are frequently used in dentistry to address full and partial edentulism. In comparison to traditional fixed partial dentures, dental implants offer several advantages, such as a high success rate (over 97% over 10 years), a decreased risk of caries and endodontic issues in neighbouring teeth, improved bone maintenance in the edentulous site, and reduced sensitivity of neighbouring teeth [1]. A fixed or removable tooth prosthesis is retained and supported by a dental implant, an alloplastic structure inserted into the oral tissues beneath the mucosa and periosteum, as well as within or through the bone. Around 600 AD, Mayans used shell pieces as implants to substitute mandibular teeth in patients. Maggiolo implanted a gold conduit into a fresh extraction site in 1809. The Strock siblings replaced missing teeth with Vitallium screws in 1930 [1]. In the 1940s, Formiggini and Zepponi-the founders of contemporary implantology-created the first post-style endosseous implant. In the 1940s, Dahl began the subperiosteal transplant in Sweden [2]. Strock made a two-stage screw implant without a per mucosal post in 1946. The individual crown and abutment post were affixed after the implant had fully healed. At the time, the intended implant interface was referred to as ankylosis. Blade implants, also known as endosseous implants, were created by Dr. Linkow in 1967. Due to Dr. Branemark's unintentional finding, which helped advance the concept of osseointegration (direct, rigid connection of the implant to the bone without any intervening tissue between two implants), dental implants gained popularity [1].

“Osseointegration was originally defined as a direct structural and functional connection between ordered living bone and the surface of a load-carrying implant.” If there is no progressive relative movement between the implant and the bone it is in close contact with, it is said to have osseointegrated. Although titanium metallic implants were the subject of "osseointegration" at first, it is now used to refer to any biomaterial that can osseointegrate. The osseointegration process is closely related to biomaterials, designed to be implanted or incorporated into living systems to replace or regenerate tissues and tissue functions [1,2].

“The periodontal ligament (PDL), a fibrous network, tightly connects both the tooth root's cementum and the mandible's alveolar bone.” The alveolar bone and tooth are shielded from the high pressures of mastication by their mechanical stability and shock absorption. The PDL and gingiva also work together to create a barrier that protects the oral area from pathogens. Last but not least, the sensory information in the mastication system is greatly influenced by the neural network inside the PDL. These abilities deteriorate in periodontitis, a common and aggravating disease that causes the loss of the PDL and the supporting alveolar bone, damaging the PDL [3]. Periodontitis left untreated ultimately results in tooth loss. Although there are periodontitis treatments accessible, their primary focus is on symptom management and disease progression inhibition rather than tissue regeneration. Given the high rate of periodontitis recurrence and the anticipated rise in patients with periodontal disease as the population ages, newer therapeutic measures targeted at tissue regeneration are urgently required. Collagen fibres, primarily type I collagen, provide structural strength to the PDL, with a minor contribution from collagen type III [2,3].

The health sciences are home to the rapidly expanding interdisciplinary discipline of tissue engineering (TE), which is focused on imitating natural biological development to create intact tissue and organ constructs. The field works with cells, organic or synthetic scaffold materials, and bioactive molecules to regenerate entire biological components outside the body (i.e., implantation) [4]. All surgical subspecialties need viable, transplantable body parts for reconstructing anatomical structures, and TE can fill that need. By acting as a potentially infinite source of tissues and organs and avoiding the need for immunosuppression through the expansion of autologous cells, TE has demonstrated the potential to address essential issues in transplantation, such as the shortage of donor organs and the requirement for lifelong immunosuppression [4].

PDL tissue engineering (TE) has multiple unique challenges. First, off-the-shelf constructs are constrained by the exceedingly small PDL space, which measures 150 and 400 mm from the alveolar bone to the tooth. The second challenge is making and accurately anchoring a soft tissue between two mineralised surfaces. Keeping a line between teeth and bone is challenging enough to stop mineralisation from progressing [3,4]. Third, since damage brought on by high forces is almost always inevitable, PDL engineering constructs must be functionally capable of withstanding high forces and include a self-repair mechanism to guarantee their integrity. As a result of these forces, natural PDL fibres align with the size and direction of loading, enhancing that direction's mechanical strength. Aligning structures used in tissue engineering can be done in several ways. For PDL engineering, many different polymers have already been suggested. These include synthetic polymers, hydrogels, glasses, ceramics, and natural polymers [3]. This review section aims to highlight the advantages of periodontal ligament-engineered implants over conventional implants and the processing, benefits, success, and future perspective of the ligaplant.

## Review

Search methodology

**Figure 1:**
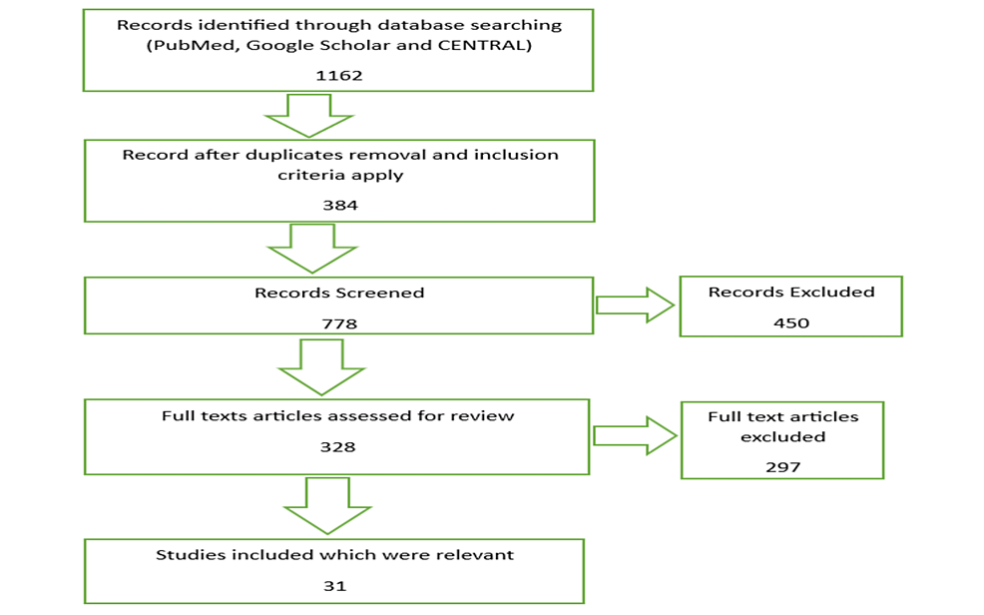
PRISMA Flowchart of the Review. Adopted from the Preferred Reporting Items for Systematic Reviews and Meta-Analyses (PRISMA).

We searched PubMed, Google Scholar, and Cochrane Central Register of Controlled Trials (CENTRAL) from October 2022 using keywords ligaplants, PDL, Osseointegration, Tissue-engineered ligament, Implants, Tissue engineering, and biomaterial. Also, we checked the articles based on the title and abstract before the full text. We have curated articles that included methodology, studies, and case reports regarding the concept of ligaplant, its success, advantages and disadvantages, properties, and clinical aspects. We excluded 450 articles which were included tissue engineering in oral dentistry and reviewed 328 articles that included tissue engineering related to implant dentistry and periodontal ligament. Figure 1 details the PRISMA flow diagram for the same.

What are ligaplants

The combination of PDL cells with implant biomaterial is known as ligaplants. Currently, implants are made of inert biomaterials placed straight into the jawbones to replace missing teeth, disregarding the PDL. In cases of gingival recession, where the altered tissue architecture may require additional surgical interventions, a localised bone loss around the implant fixture presents a clinical challenge. An implant device with tissue-inducing capabilities might be helpful to solve this issue [5]. Technically, PDL-engineered implants can be positioned in the extraction socket of a lost tooth, simplifying surgery. Future development may also be compatible with natural implant anchoring. Last but not least, ligaplants can stimulate the growth of new bone when positioned in locations linked to significant periodontal bone defects. PDL implants are inserted into the extraction socket of the missing tooth, simplifying the surgical process [5,6].

**Figure 2:**
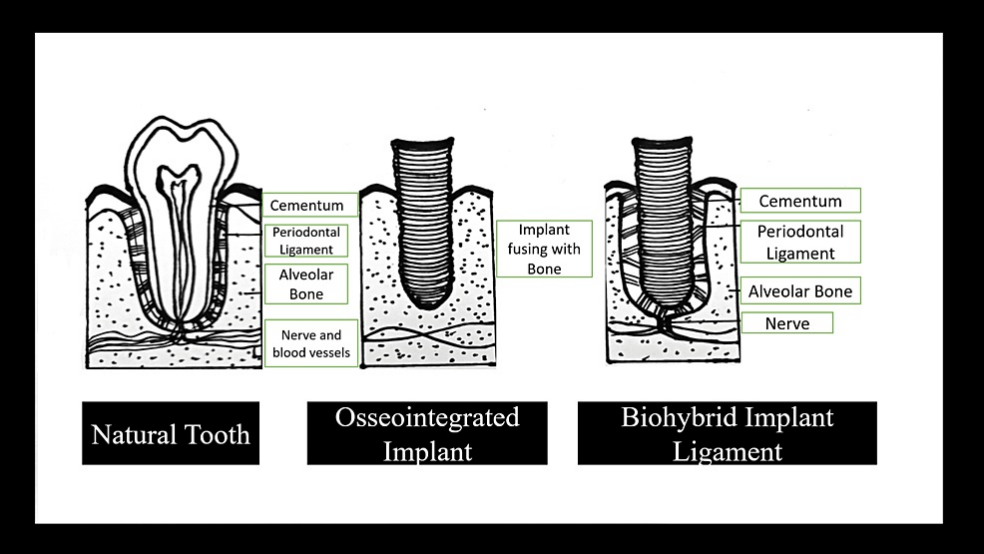
A diagram comparing a natural tooth, an osseointegrated implant, and a ligaplant. Created by Author

Due to their high long-term clinical survival rate, osseointegrated implants are the most acceptable. If a PDL implant could be created, ligaplant, a combination of PDL cells and implant biomaterial, could address these problems. Because they are "ankylosed," osseointegrated implants lack the same movement as natural teeth with a PDL. Years ago, "shock-absorbing systems" integrated into the implant or its superstructure were attempted to make up for this glaring disparity [6]. "The ligaplant's combination of periodontal ligament cells and implant biomaterial produces periodontal ligaments that allow for micro-movements and shock absorption, which enhances the quality of the force distribution among the teeth abutments and prosthetics supported by the implants.” These implants may be used to improve physiological outcomes and address the limitations of conventional implants, possibly extending the prosthesis's lifespan. They resemble the appearance and function of natural teeth [7]. Figure 2 compares a natural tooth, an osseointegrated implant, and a ligaplant.

Characteristics of the ligaplants

Ligaplants promote sensory perception or the sense of one's movement and position. It helps to distribute occlusal and masticatory forces. In contrast to conventional implants, they provide shock absorption capacity. Different types of undifferentiated cells provide the osteoconductive properties of the implant. It provides natural-dentition-like anchoring, allowing for tooth movement during orthodontic treatment. ligaplant offers sufficient anchoring to support the growth and development of alveolar bone housing [8].

Tissue Engineering

With numerous advancements in various disciplines, tissue engineering technology is thriving and has emerged as a well-liked study technique for reconstructing damaged or missing tissues. Therefore, the development of tissue engineering technology will significantly advance stomatology. The fundamental principle of tissue engineering is to gather functionally related cells, place them on a natural or artificial scaffold with a predetermined spatial arrangement, and then use growth factors to stimulate cell proliferation, leading to tissue regrowth [8]. “Periodontal tissue engineering is a novel idea for reconstructing damaged periodontal tissues and organs that have seen rapid growth in recent years, in contrast to conventional periodontal therapy.” Traditional tissue engineering methods rely on scaffolding materials and seed cells. Although dental implants eliminate some of the disadvantages of dentures and effectively repair defects caused by tooth loss, two factors continue to hinder dental implant technology advancement: first, insufficient local bone mass in the implants, and second, inadequate soft tissue around the implants. Dental implant tissue engineering is primarily concerned with modifying the alveolar bone and soft tissue environment before implant placement in the edentulous area to achieve good osseointegration and soft tissue augmentation [4,8,9].

Properties

The tooth may move within the socket as a shock absorber. Additionally, proprioception is offered as well. The PDL also interacts with the nearby bone significantly, serving as the periosteum on the side of the bone that faces the root. Ligaplants contain essential cells like immature mesenchymal stem cells, cementoblasts, cementoclasts, osteoclasts, fibroblasts, and cementoblasts. Each cell contributes to the dynamic interaction between the bone and the teeth [10].

Advantages and disadvantages

The advantages and disadvantages of ligaplant are detailed in Table 1. 

**Table 1:** Advantages and disadvantages of the tissue-bioengineered ligament. [11-13]

Advantages	Disadvantages
It addresses problems like bone defects and gingival recession brought on by lost teeth.	Care should be taken when performing ligaplant culture. For instance, the temperature, the type of cells used for growing, how long it took, and so forth. The development of nonperiodontal cells may cause the ligaplants to fail if a problem occurs during the culturing procedure.
Simulates natural tooth root insertion in the alveolar process.	The price of this implant is also high due to a need for more facilities.
Ligaplants initially fit loosely to protect the PDL cell cushion, but they eventually become firmly integrated without locking or direct bone contact.	The growth of PDL in the socket or the variables affecting the host's acceptance of the implant are unpredictable, which could lead to implant failure.
In the transmission of chewing forces from bone to teeth.	Unpredictability in host acceptance.
Its ability to remodel bone (the presence of the PDL maintains/regenerates bone quality)	The procedure is expensive.
Promotes new bone formation even when placed in areas with significant periodontal defects, eliminating the need for bone grafting [11-13].	Cell culture for an extended time may favor the appearance of non-PDL cell types [11-13]

Clinical considerations

**Figure 3:**
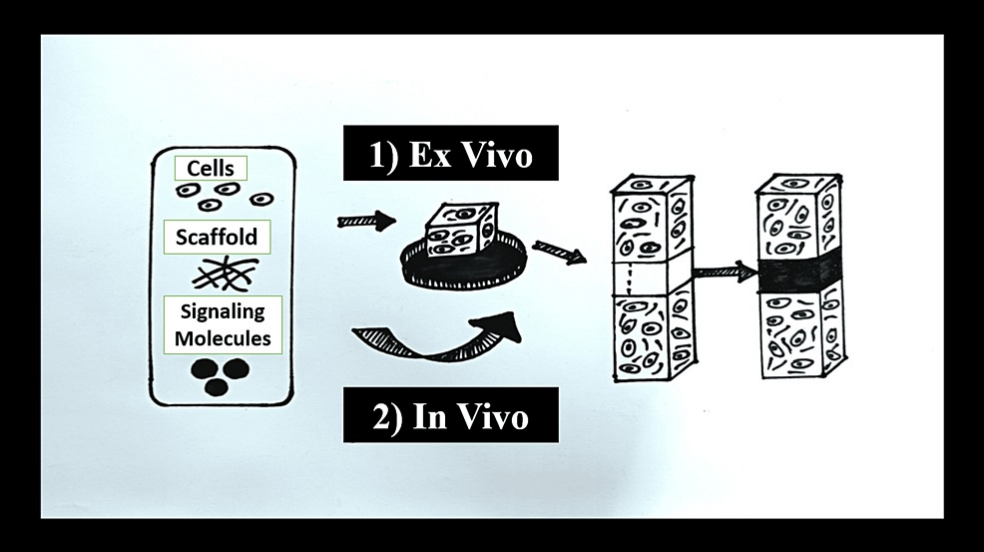
Ex vivo and in vivo techniques for reconstruction (1) Ex vivo technique- to grow tissue or organs in a culture chamber using the three components support or matrix, signaling molecules, and cells before implanting tissue-engineered organs into patients. (2) In vivo technique- uses the three components to stimulate intrinsic healing activity at the location of the tissue defect. Created by Author

The essential elements for reconstruction and regeneration include a matrix or scaffold, molecular messengers, and cells. In vitro cultures are created from laboratory-made tissues. Before being transplanted into the body, cells are grown on biodegradable scaffolds or matrices with signalling molecules [6]. The in vivo method is applied when all of the developed vital components are inserted into a tissue defect and allows the body's natural healing process to take place, resulting in regeneration. The tissue defect location experiences intrinsic healing activity by combining the three components. Both in vitro and in vivo methods can achieve this. Figure 3 shows the Ex-vivo and In-vivo techniques [6,14].

The triumph of the ligaplants

Site-specific signaling, which is required for the growth of a regenerating PDL, is carried out by transcription factors with homeogene-coded expression patterns that carry anatomical information. The synthesis of cell surface and signaling components is influenced by homeoproteins, and signals from cell surface feedback modulate the expression of homeogenes, resulting in cell identities that are defined by anatomic location and tissue type [15].

Risk factors associated with ligaplants

Periodontal ligament development depends entirely on on-site signaling, mainly controlled by anatomical code and transcription factors with homeogene-coded DNA. The result of communication elements and the cell surface depends on these homeoproteins. Since variables affecting the required site's growth of periodontal ligaments are typically unknown, they pose a risk for treatment outcomes [7]. The medium flow should have slight mechanical movements while setting up the ligaplant. The ideal distance between the device and the culture is needed. For the best opportunity of getting a successful ligaplant, the time spent on surface treatment should also be optimal. Additional sanitation is required. Optimising cell culture will stop nonperiodontal ligament cells from growing [12].

Surgical procedure

Temperature-Responsive Culture Dish Preparation

Polystyrene culture plates were exposed to a solution of 2-propanol and nisopropylacylamide monomer. The Area Beam Electron Processing System was then used to bombard these plates with an electron beam. The dishes were washed in cold water, rinsed, and sterilised with ethylene oxide. They were temperature-responsive polymer-grafted with (N isopropyl acrylamide) [16].

Cell Isolation and Culture

An extracted tooth is used as the source for the isolation of human periodontal cells. After extraction, A scalpel blade removes periodontal tissue from the middle third of the root. The tissue is placed in culture dishes containing Dulbecco's modified Eagle's minimum essential medium, 100 units/mL of penicillin-streptomycin, 10% foetal bovine serum, and other ingredients [17]. After 48 hours of culture, the outgrowth cells are allowed to adhere to the plates in a humidified environment of 5% CO_2_ at 37°C. The medium is replaced thrice weekly, and the dishes are washed to eliminate debris. An osteo-differentiation medium comprising 50mg/mL ascorbic acid 2-phosphate, 10nM dexamethasone, and 10nM glycerophosphate is used to culture human periodontal ligament cells, which are plated at a cell density of 1x105 on temperature-responsive culture dishes (35 mm in diameter) [16,17].

PDL Cell Culture in a Bioreactor

A plastic cylinder with a hollow interior and a 3mm gap on all sides contained a titanium pin covered in hydroxyapatite (HAP). The culture medium was continuously poured into the gap. First, plastic containers are seeded with the human single-cell suspension (periodontal ligament cell suspension) for 18 days [16,17].

Future aspects

The future of implant dentistry will be tissue-engineered ligament-equipped implants, which beat conventional implants today. More clinical studies, especially in humans, are required to determine these implants' long-term stability, functionality, survival, and success because a predictable and practical method for creating dental implants with periodontal-like ligaments has yet to be developed. Most of these studies were conducted on animals, and they demonstrated that it is possible to create periodontal-like tissue around implants [8,13].

Discussion

To accomplish osseointegration, dental implants are placed. The redevelopment of PDL around the device has yet to be considered in this process. Osteointegrated implants affixed straight to the neighbouring alveolar bone do not serve the same purpose as natural teeth because the natural periodontal tissue is absent. Therefore, it is crucial to construct a well-designed dental implant in conjunction with a tissue-engineered PDL, as this establishes a biological connection capable of performing specific physiological functions. Periodontal ligament cells can restore connective tissue attachment to the surface of the teeth, according to 1982 research by Nyman et al. In 1990, Buser et al. concluded that titanium implants used to replace retained root tips had a cementum layer and PDL development on their surface [6,18-21]. Periodontal ligament cells were placed in the alveolar process after being cultured on a titanium pin's surface, as Gault et al. reported in 2010 [17]. The alveolar bone and titanium pins were integrated, and new bone grew nearby [18]. Dental progenitor cells on dental implants showed the presence of PDL on the surface of dental implants, which was confirmed in a 2011 study by Lin et al. [19]. In their research, Kiong et al. (2014) noted that ligaplants are a tooth replacement option with significant benefits over dental implants and relatively simple surgery [20].

In 1982, Nyman et al. proposed that PDL cells can restore connective tissue attachment [21]. In research, Nunez et al. 2012 further demonstrated the regenerative capacity of PDL-derived cells. Numerous in vitro studies show that when dental implants are positioned near the radicular portion of the tooth, cementum-like tissue with an intervening PDL forms. Although partial regeneration of the periodontium, consisting of cementum, PDL, and alveolar bone, was feasible, technical and physical barriers made using such techniques in patients seem impractical [22]. To create human tooth-ligament interfaces, Park et al. have demonstrated potential for the therapeutic application of specialised periodontal biomimetic hybrid scaffolds [23]. In animal research, Takata et al. looked into the possibility of connective tissue attachment on implant materials using PDL-derived cells. They found that while cementum deposition was little to nonexistent on bioinert materials like titanium alloy and partly stabilised zirconium, new connective tissue attachment occurred on bioactive materials like bioglass and hydroxyapatite. This suggests that bioactivities impacted the development of novel connective tissue attachment [24]. Choi BH implanted cultured autologous PDL cells into the dogs' mandibles, and histological analysis showed that after three months of healing, some implant surfaces had developed a layer of cementum-like tissue with insertions of collagen fibres. This demonstrated the ability of cultured PDL cells to create tissue around implants that replicate a real PDL [25]. Kano et al. hypothesised that implants surrounded by PDL-like tissue may develop when tooth-shaped titanium implants with hydroxyapatite coatings are inserted into the tooth socket where some PDL is still present right away after the extraction; the preservation of original periodontal tissue domains is most likely what prevents the implants from osseointegrating [26].

By incorporating PDL stem cells (PDLSCs), recent research has improved the safe and successful usage of cell sheet engineering to replace the cementum around dental implants [27]. Therefore, a mix of bioengineering methods improved with appropriate adult stem cells as a possible candidate for regenerative therapies combined with current dental implant design is the research strategy for the future. Future clinical applications of artificial PDL could include the effective restoration of perception through suitable nerve innervations, regeneration of periodontal tissue, and reduction of the requirement for bone grafting in periodontal bone abnormalities [28]. Because of their distinct wound healing mechanism and rich vascularity and cellular defence system, these hybrid live dental implants can be used in clinical situations requiring periodontium repair and in areas vulnerable to infections where osseointegrated implants are not suggested [29-31].

## Conclusions

Implant dentistry has reached a new level due to periodontal ligament tissue engineering. Ligaplants increase stability over a long period by promoting the best performance of healthy human teeth with minimal inconvenience and discomfort. Ligaplant is a new tissue engineering application that researchers and clinicians are exploring. Newer tissue is consistent with PDL that forms on the surface of dental implants after implantation and has significant benefits over osseointegrated dental implants because of periodontal tissue regeneration.
